# The role of miRNAs as biomarkers in breast cancer

**DOI:** 10.3389/fonc.2024.1374821

**Published:** 2024-05-15

**Authors:** Temesgen Baylie, Mulugeta Kasaw, Mamaru Getinet, Gedefaw Getie, Mohammed Jemal, Amare Nigatu, Hassen Ahmed, Mihiret Bogale

**Affiliations:** ^1^ Department of Biomedical Science, School of Medicine, Debre Markos University, Debre Markos, Ethiopia; ^2^ Department of Biochemistry, School of Medicine, College of Medicine and Health Sciences, Bahir Dar University, Bahir Dar, Ethiopia; ^3^ Department of Biochemistry, School of Medicine, College of Medicine and Health Sciences, Woldia University, Woldia, Ethiopia; ^4^ Department of Biochemistry, School of Medicine, College of Medicine and Health Sciences, Wollo University, Wollo, Ethiopia

**Keywords:** BC, miRNA, circulating biomarker, diagnosis, prognosis, therapy, metastasis

## Abstract

Breast cancer (BC) is the second most common cause of deaths reported in women worldwide, and therefore there is a need to identify BC patients at an early stage as timely diagnosis would help in effective management and appropriate monitoring of patients. This will allow for proper patient monitoring and effective care. However, the absence of a particular biomarker for BC early diagnosis and surveillance makes it difficult to accomplish these objectives. miRNAs have been identified as master regulators of the molecular pathways that are emphasized in various tumors and that lead to the advancement of malignancies. Small, non-coding RNA molecules known as miRNAs target particular mRNAs to control the expression of genes. miRNAs dysregulation has been linked to the start and development of a number of human malignancies, including BC, since there is compelling evidence that miRNAs can function as tumor suppressor genes or oncogenes. The current level of knowledge on the role of miRNAs in BC diagnosis, prognosis, and treatment is presented in this review. miRNAs can regulate the tumorigenesis of BC through targeting PI3K pathway and can be used as prognostic or diagnostic biomarkers for BC therapy. Some miRNAs, like miR-9, miR-10b, and miR-17-5p, are becoming known as biomarkers of BC for diagnosis, prognosis, and therapeutic outcome prediction. Other miRNAs, like miR-30c, miR-187, and miR-339-5p, play significant roles in the regulation of hallmark functions of BC, including invasion, metastasis, proliferation, resting death, apoptosis, and genomic instability. Other miRNAs, such as miR-155 and miR-210, are circulating in bodily fluids and are therefore of interest as novel, conveniently accessible, reasonably priced, non-invasive methods for the customized care of patients with BC.

## 1 Introduction

Worldwide, Breast cancer is a significant public health concern that impacts women. It is the most common type of cancer diagnosed worldwide and the second leading cause of cancer-related deaths in women (1). In 2020, there were *2.3 million* women diagnosed with BC and 685 000 deaths globally. Approximately 0.5–1% of BCs occur in men (2). When BC spreads (metastasizes) to other parts of the body, it becomes the most severe type of the disease, greatly increasing the tumor burden and frequently leading to a fatal diagnosis (3). BC metastasis is a cascade that begins with localized tissue invasion, progresses into blood or lymphatic vessels, and culminates in tumor cell dispersion to distant organs (4). The progression of the primary cancer, angiogenesis, cancer cell invasion, lymphatic/blood vessel invasion, extravasation into a distant organ, and colonization and growth of metastatic tumors are all included in this process (5). Up to 5% of individuals with metastatic BC (MBC) present with distal metastases at the time of diagnosis, making the disease frequently incurable even with current treatment options. Nowadays, 10–15% of individuals develop distant metastases (M1) throughout the first three years. Moreover, about one-third of BC patients who do not have lymph node involvement at the time of diagnosis will experience distant metastases (6). Importantly, the initial tumor subtype is likely to have an impact on the rate and place of metastasis, which can vary significantly. It is obvious that further research is required in order to properly diagnose and treat metastatic BC. MmiRNAs have demonstrated potential as novel biomarkers for many malignancies, such as metastatic BC, in recent times (7, 8). Importantly, miRNAs have been linked to all stages along the metastatic cascade in BC (9). This review covers the clinical potential of miRNAs as biomarkers of early diagnosis and prognosis, as well as determinants of metastasis and therapy. It also summarizes current articles on miRNA activities in the regulation of BC progression. A review of some BC therapeutic approaches including miRNAs offers fresh perspectives on the treatment of BC.

## 2 Biogenesis of miRNA

**Figure 1:**
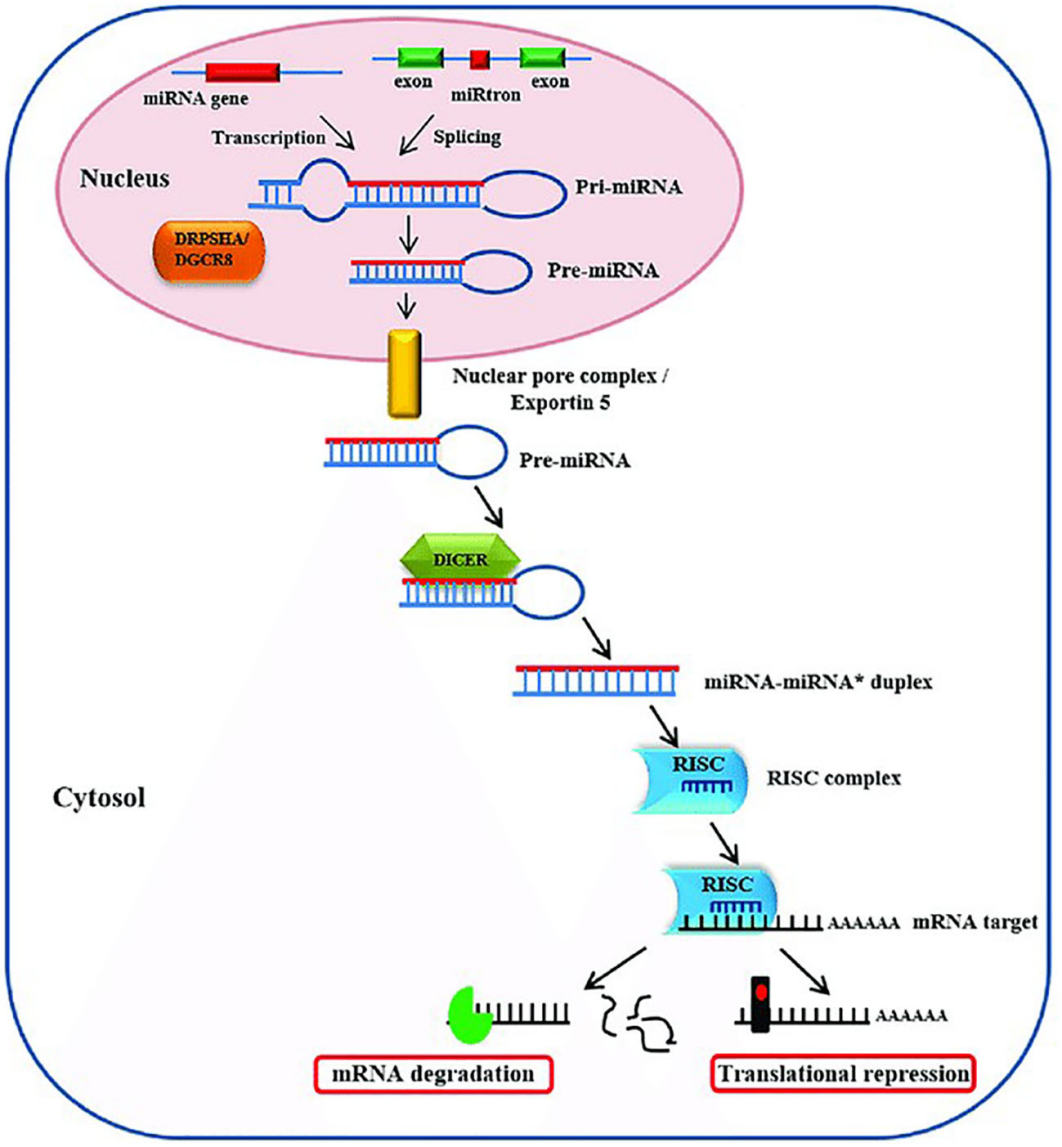
Human cells use the nucleus as the starting point for the multistep process of miRNA synthesis and processing. Here, the RNase III enzyme Drosha, along with its binding partner DGCR8, cleaves nascent miRNA transcripts, or pr-miRNA, into about 70 nucleotide precursors, or pre-miRNA. These pre-miRNAs’ stem-loop structure isn’t the best. Pre-miRNAs are subsequently exported by exportin 5 from the nucleus into the cytoplasm. The hairpin precursors are degraded by Dicer and its binding partner, the trans activator RNA-binding protein TRBP, creating a tiny, incomplete dsRNA duplex (miRNA:miRNA*) in the cytoplasm. The mature miRNA strand and its complementary strand are both present in this duplex. Targeting complementary mRNA regions, the miRNA strand is incorporated into the miRNP complex and functions through translational repression or mRNA cleavage.

The primary transcript known as pri-miRNA, which has a 33 bp hairpin stem, a terminal loop, and a flanking single-stranded sequence of hundreds of bases or even several kilobases, is the first step in the biogenesis of miRNAs. Some other miRNAs, however, are transcribed by RNA polymerase III. Pri miRNAs are typically polyadenylated at the 3´end and capped at the 5´end (10). There are multiple post-transcriptional processing stages needed to produce the mature, functioning miRNA. The Drosha enzyme, which is linked to the DGCR8 (DiGeorge Syndrome Critical Region 8) (**Figure 1**) protein, detects the stem loop formed by pri-miRNA and converts it into a double-stranded (ds) hairpin shape that is roughly 70 nucleotides long and is known as precursor miRNA (pre-miRNA) (11). The nucleocytoplasmic protein Exportin-5 is involved in the process by which pre-miRNA hairpin is exported from the nucleus. The RNase III enzyme Dicer cleaves the pre-miRNA hairpin in the cytoplasm, creating a miRNA duplex of 18–22 nucleotides. (12). The RNA-induced silencing complex (RISC), which is the site of interaction between the miRNA and its mRNA target, typically incorporates only one duplex strand, despite the possibility that both strands could function as miRNAs. Mature miRNA functions by either preventing the translation of the mRNA target or by deteriorating it. A degraded dsRNA strand is typically referred to as miRNA*, while the mature single-stranded (ss) miRNA on the other hand forms the miRNA-associated RNA-induced silencing complex (miRISC) (13). By attaching to the 3′-untranslated region (3′-UTR) of the target mRNA, miRNA in miRISC has the potential to affect gene expression. While miRNA has a region of 2–8 nucleotides at its 5′ end known as the seed region, which controls its specificity and attaches to mRNA at its 3′-UTR, this area is made up of sequences that miRNA recognizes (14).

## 3 Regulatory miRNAs in the development of BC

**Figure 2:**
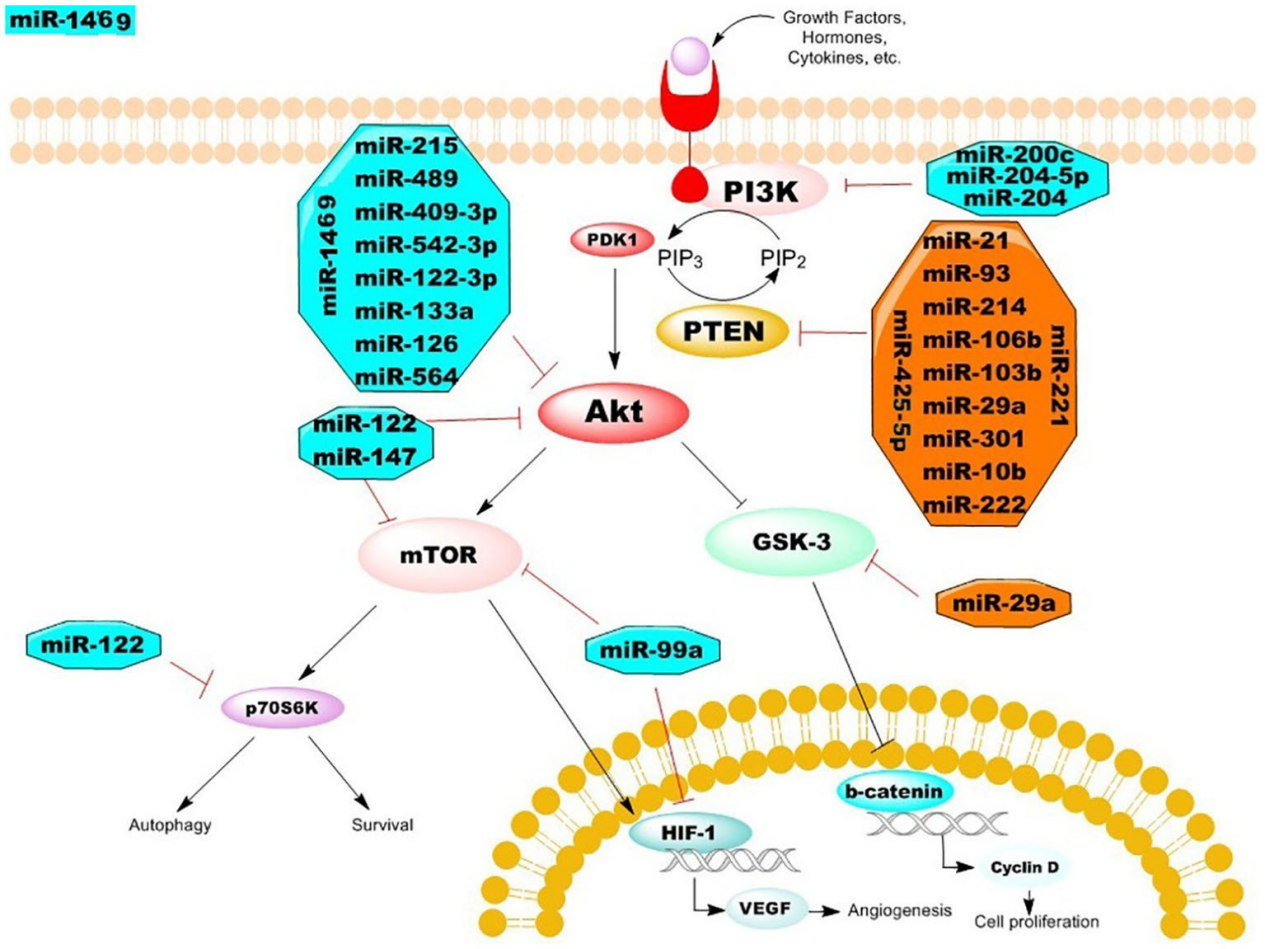
The mechanisms of PI3K/AKT/mTOR regulatory miRNAs in the development of BC.

A key intracellular signaling axis, PI3K signaling combines several signals to promote the growth and spread of cancer cells. The three primary elements of the signaling pathway are mTOR (mammalian target of rapamycin), AKT (protein kinase B; PKB), and PI3K (phosphatidylinositol-3-kinase) (15). Phosphatidylinositol 4,5-bisphosphate (PIP2) is phosphorylated by the PI3Ks, a family of kinase enzymes, to produce phosphatidylinositol 3,4,5-triphosphate (PIP3) (**Figure 2**). The phosphorylation of AKT, a master regulator that can directly affect multiple downstream targets like the mTOR and WNT/β-catenin pathways, is encouraged by this PIP3 synthesis. AKT has the ability to control angiogenesis, apoptosis, metabolism, and cancer cell growth (16). AKT activates the mTOR complexes, which control a number of processes related to cell development, such as protein synthesis, cell survival, and autophagy inhibition. Many human cancers, including BC, commonly activate PI3K signaling, and persistent activation of PI3K signaling is associated with a poor prognosis and resistance to chemotherapy (17).

## 4 Oncogenic MiRNAs in BC

Certain miRNAs function as Oncogenic and are expressed more frequently in BC. These miRNAs decrease the expression of antioncogenes in apoptosis, metastasis, invasion, and cell proliferation. The miR-10, miR-15, miR-16, miR-17~92 cluster, miR-18, miR-19, miR-20, miR-21 family, miR-92, miR-155, and miR-569 have been identified as the carcinogenic miRNAs and their families (18).

The miR-10 family includes subtypes called miR-10a and miR-10b, which are involved in development and metastasis. The miR-10 family has a role in development by controlling Hox transcripts. While miR-10b expression levels are adversely connected with E-cadherin, they also increase tumor growth, metastasis, and clinical stage. In a mouse xenograft model of BC, it was found that overexpression of miR-10b promotes invasion and migration (19).

Together with the transcription factor E2F1, miR-17 affects the cell cycle and promotes the progression of cancer. In lymphomas, the miR-17~92 cluster is amplified. Despite the fact that this miRNA cluster was found to be downregulated in metastasis, miR-17-5p was not like the others (20). In invasive MDA-MB-231 BC cells, it is expressed at very/extremely high levels, but not in non-invasive MCF-7 BC cells. Targeting the HBP1/β-catenin pathway, this group can induce migration in MCF-7 cells. *In vitro*, the invasion of MDA-MB-231 cells is suppressed by reducing miR-17-5p (21).

**Figure 3:**
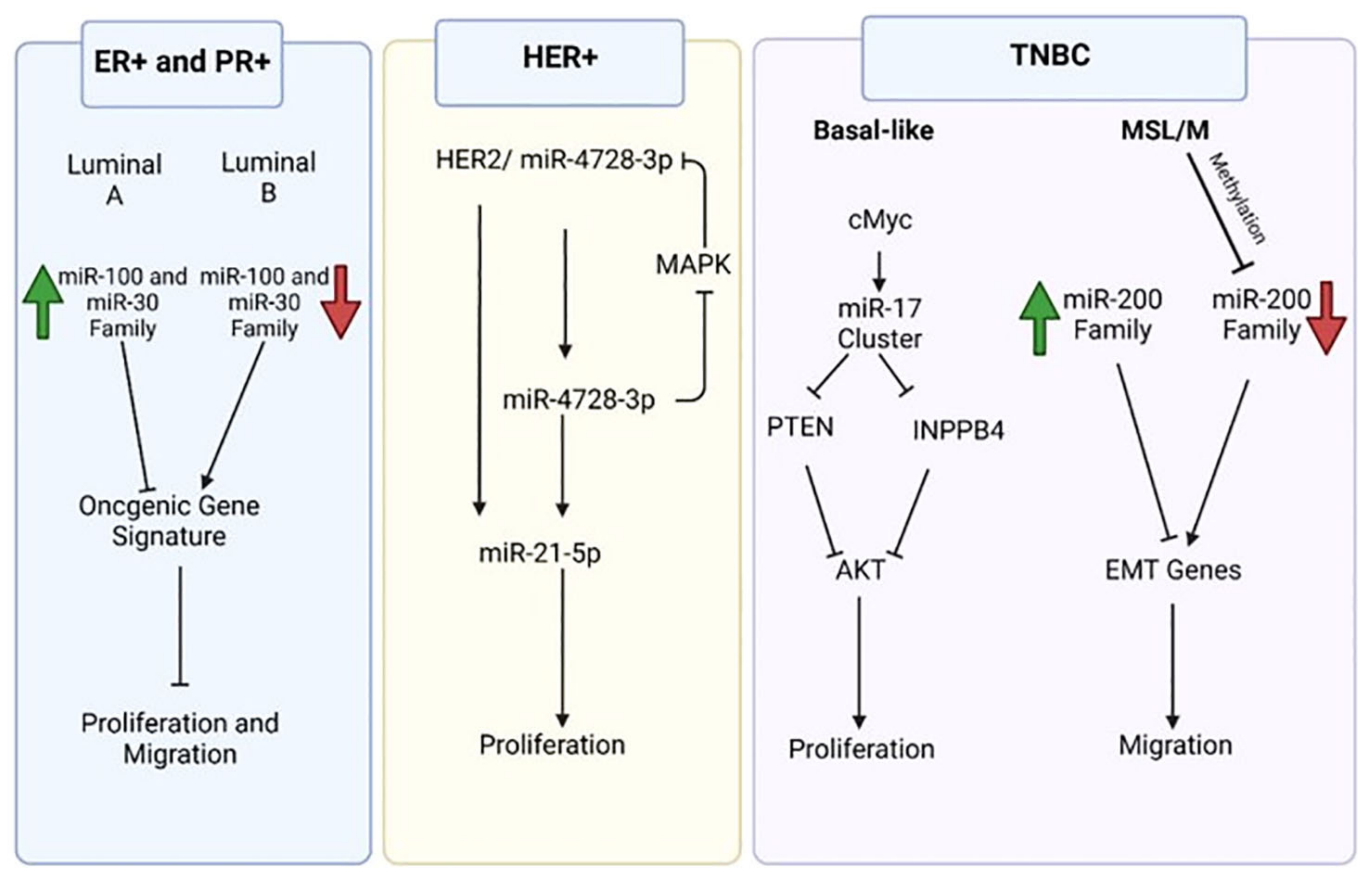
Molecular signaling mediated by miRNAs networks specific breast cancer subtype.

In PI3K/AKT signaling, aberrant expression of S regulatory proteins led to unchecked cell division and BC carcinogenesis. Master regulators like phosphatase and tension homolog (PTEN) are targets of several miRNAs that can stimulate the PI3K/AKT signaling pathway (22). By dephosphorylating PIP3, the phosphatase enzyme PTEN inhibits the activity of PI3K. PTEN was discovered to be the target of multiple miRNAs, such as miR-21, which is overexpressed in triple negative and HER2+ (human epidermal growth factor receptor 2-positive) BCs, leading to the advancement of the tumor (**Figure 3**) (23). Furthermore, by directly targeting PTEN and inhibiting PI3K/AKT signaling, miR-21 prevents the process of autophagic cell death (24). As a result, targeting miR-21 or upregulating PTEN may be useful therapeutic approaches for BC. Furthermore, it has been observed that miR-93, miR-301, and miR-106b control PTEN expression, which leads to inappropriate activation of PI3K/AKT signaling (25). According to recent research, individuals with BC have higher expression levels of miR-93, miR-301, and miR-106b, which promotes the growth and invasion of tumor cells. Zhang et al. demonstrated that miR-425-5p can directly target the 3′UTR of PTEN mRNA, resulting in downregulation of PTEN both in mRNA and protein levels, further supporting the regulatory influence of miRNAs on PTEN expression. Furthermore, it has been demonstrated that forced expression of miR-425-5p promotes the development and metastasis of BC cells by over-activating PI3K and AKT enzymes and upregulating cyclin proteins (26). Furthermore, compared to healthy controls, BC patients’ serum levels of miR-214 were noticeably greater. The results of ROC analysis suggest that miR-214 levels can differentiate between benign and malignant tumors. Furthermore, it has been demonstrated that in individuals with BC, blood levels of miR-214 were strongly connected with tumor metastasis and poor outcomes (27). By directly targeting PTEN expression, miR-214 promotes PI3K signaling, which in turn causes BC to develop and spread. In addition, patients’ overexpression of miR-19b was noted when contrasted with normal controls. It’s interesting to note that cellular migration and proliferation have been found to be positively correlated with miR-19b overexpression causing the spread and metastasis of cancer (28). According to the survival study results, miR-19b expression was associated with a worse overall survival, indicating that it may have predictive value for patients with BC. Furthermore, it has been demonstrated that the expression of MiR-10b is linked to the metastasis and survival of BC cells. It has been discovered that the overexpression of epithelial-mesenchymal transition (EMT) markers in BC is closely correlated with the prolonged expression of miR10b. Antisense RNAs that inhibited miR10b caused PTEN to be up-regulated and AKT to be suppressed (29). Additionally, it was found that BC tissues and cell lines overexpressed miR-20b. Upregulation of miR-20b has been linked to the growth and colonization of cancer cells, according to recent research. The results of the gene expression study showed that upregulating miR-20b reduced the PTEN protein level while having no effect on the expression of its mRNA. Thus, it is possible that miR-20b’s anti-tumor action results from inhibiting PTEN protein translation in BC cells (30). Recent studies showed that drug resistance in tumor cells was caused by the overexpression of several oncogenic miRNAs. For instance, it has been found that overexpression of the genes miR-29a, miR-221, miR-222, and miR-130b causes BC and imparts resistance to Adriamycin during cancer therapy. The overexpression of miR-29a, miR-221, miR-222, and miR-130b stimulates the proliferation of tumor cells by activating PI3K/AKT and targeting several tumor suppressors, such as glycogen synthase kinase 3β (GSK3β) and PTEN (31).

A number of oncogenic miRNAs, including miR-10b and miR-21, have repeatedly been shown to be elevated in ER+ breast cancer (32). Specifically, several oncogenic miRNAs that are overexpressed in ER+ breast cancer directly target the tumor suppressor PTEN, which dephosphorylates PIP3 and subsequently suppresses PI3K/Akt signaling (**Figure 3**). Therefore, the abnormal overexpression of oncogenic miRNAs in ER+ breast cancer attenuates PTEN-mediated inhibition of PI3K/Akt signaling, which in turn increases oncogenic cell growth, survival, and migration through downstream Akt signaling (33). For patients with ER+ breast cancer, targeted inhibition of these oncogenic miRNAs may offer additional therapeutic alternatives. The control of estrogen/ERα signaling is largely dependent on miRNAs, and ERα activation can also modify numerous miRNAs (34).

## 5 Tumor suppressor MiRNAs in BC

The let-7 families are the most ancient and conserved miRNAs. They perform the role of well-known tumor suppressive miRNAs in a heterochronic system required for the proper initiation, differentiation, and metastasis of malignancies at the proper times, including BC (35). When a breast tumor is first developing, let-7a silences its target genes, HMGA2 (high-mobility group AT-hook 2) and H-Ras (transforming protein p21), in order to perform BT-IC stem cell-like functions. The development of BC is substantially postponed by let-7 overexpression. suggested that let-7b/c down-regulation in BC stem cells led to let-7b/c losing its capacity to suppress Ras mRNA, which activated p-Ras and p-ErK (36). This demonstrated that let-7 plays a critical function in preserving the stemness of BC cells. BC cells’ lymph node metastases frequently exhibit down-regulated let-7b. Breast migration and invasion are significantly reduced through the silencing of four actin cytoskeleton pathway target genes: ITGB8 (integrin β–8), RDX (radixin), DIAPH2 (protein diaphanous homolog 2), and PAK1 (serine/threonine-protein kinase 1) (35).

The five subgroups that constitute up the miR-200 family are miR-200a, miR-200b, miR-200c, miR-141, and miR-429. EMT is suppressed by the miR-200 family through the control of E-cadherin. A moderating influence of miR-200 families is seen in the regulation of the change from CSC-like to non-stem-cell-like characteristics (37). Epigenetic changes under stem-like phenotypes resulted in the silencing of the miR-200b-200a-429 cluster and the suppressed expression of the miR-200c-141 cluster. Additionally, miR-200 families have moesin-dependent effects at various phases of metastasis (35). As a tumor suppressor, miR-200b can control the metastasis and plasticity of tumor cells. By preventing ZEB2 (zinc finger E-box-binding homeobox 2) expression, miR-200b/c stimulates the mesenchymal-to-epithelial cell transition (MET) and increases macroscopic metastases in BC cell lines (38).

MiR-99a has been shown to target MTOR signaling, which can prevent the growth of BC by targeting the mTOR/HIF1-α signaling pathway. In BC stem cells, upregulation of miR-99a induces cellular death, suggesting that miR-99a may function as a predictive biomarker for BC (39). It has also been found that BC is associated with downregulated miR-122 expression. Through inducing G1 cell cycle arrest, miR-122, a crucial regulator of PI3K signaling, suppresses the growth of tumor cells. These findings suggest that miR-122’s anti-tumor action might involve inhibiting the AKT/mTOR/p70s6k signaling axis. Reportedly, downregulation of miR-122-3p in BC cells is linked to increased metastasis and survival of tumor cells (40). Mechanistically, by targeting PI3K and EMT-related proteins such PTEN, AKT, Vimentin, and E-cadherin, overexpression, miR-122-3p causes cellular death while inhibiting cancer cell invasion. Moreover, there is a negative correlation between the growth of BC and miR-147 expression. BC cells had a significant suppression of miR-147 expression. By focusing on AKT/mTOR signaling, induced expression of miR-147 decreased tumor growth and metastasis (41). According to recent research, miR-200c increases the susceptibility of breast tumor cells to doxorubicin and regulates EMT and metastasis. Furthermore, it has been shown that miR-200c interacts with KRAS (Kirsten rat sarcoma) and suppresses PI3K/AKT signaling in BC via upregulating PTEN expression. By directly targeting PIK3CB, a catalytic subunit of PI3K in BC, up-regulation of miR-204-5p inhibits tumor cell proliferation and metastasis through inhibition of PI3K/AKT signaling (42). In order to delve deeper into the mechanism behind the anti-tumor action of miRNAs, Fan et al. showed that overexpression of miR-204 causes MCF-7 cells to enter a cell cycle arrest and undergo apoptosis by downregulating PI3K and AKT. Additionally, it was discovered that tissues and cells with BC have downregulated miR-1469. It has been shown that forced expression of miR-1469 suppresses the migration, invasion, and proliferation of tumor cells (43). It has been demonstrated that overexpression of miR-1469 causes cancer cell apoptosis by interrupting the cell cycle at the G2-M phase, providing more evidence for the apoptotic action of miR-1469 on MCF-7 cells. Similar to this, it was shown that BC likewise had decreased miR-409-3p expression (44). The results, both *in vitro* and *in vivo*, showed that miR-409-3p’s ectopic expression blocked the migration and proliferation of cancer cells by specifically targeting AKT1. Additionally, it was demonstrated that the tumor suppressor miR-215, which is thought to control the proliferation and invasion of breast tumor cells, targets AKT1 (45). Recent research has shown that reduced expression of miR-489 is associated with aggressive tumor features, such as metastasis and chemoresistance in BC. Furthermore, by focusing on the PI3K pathway in BC cells and tissues, it has been demonstrated that elevation of miR-489 is associated with decreased cancer cell proliferation. Moreover, apoptosis following G1 cell cycle arrest and resistance to trastuzumab were linked to significant suppression of miR-542-3p in BC (46). The discovery was made that miR-133a, which is downregulated in BC cells and tissues, targets the epidermal growth factor receptor (EGFR). One of the main tyrosine kinase receptors on the cell surface, EGFR controls the activity of multiple downstream targets, such as PI3K and AKT. According to a recent study, induced expression of miR-133a in BC targets EGFR and AKT activities while also promoting cell cycle arrest in the G2/S phase (47). It has been demonstrated that miR-133a targeted the 3′-untranslated region of the EGFR gene, reducing the expression of EGFR and its downstream targets, such as AKT. The downregulation of p-AKT protein levels caused by aberrant production of miR-133a also inhibited the nucleus translocation of p-AKT proteins. Furthermore, it has been revealed that miR-126 is downregulated in patients with BC and that it targets the VEGFA/PI3K axis to prevent the growth of cancer and angiogenesis (48). One of the key activators of PI3K/AKT signaling that promotes cancer by triggering vasculogenesis and angiogenesis is the vascular endothelial growth factor (VEGF). In an effort to better understand the regulatory role of miRNAs on the PI3K/AKT pathway, Mutlu et al. found that miR-564 inhibits the growth of tumors by encouraging G1 cell cycle arrest (49). According to more research, induced expression of miR-564 prevents BC cells from EMT and metastasizing. Through its potential dual inhibitory effect on PI3K and mitogen-activated protein kinase (MAPK) pathway components, such as AKT2, serum response factor (SRF), guanine nucleotide binding protein subunit alpha 12 (GNA12), and glycogen synthase 1 (GYS1), miR564 mechanistically suppresses the progression of breast cancer (50).

Tumor suppressor miRNAs, including miR-206, are among the several miRNAs that are downregulated in ER+ breast cancer and have the effect of inhibiting cell growth and survival. As a result, the oncogenic characteristics linked to ER+ breast cancer, such as a high proliferative index in the intrinsic Luminal B subtype of ER+ breast tumors, are facilitated by the suppression of tumor suppressor miRNAs (51). The development of targeted therapies to increase the expression of these specific tumor suppressor miRNAs holds promise for the treatment of ER+ breast cancer (52).

## 6 MiRNA as new diagnostic and prognostic markers

An early diagnosis of BC greatly increases the patient’s chances of survival compared to a late diagnosis. Various methods have been developed to find different biomarkers in order to detect BC early. In BC research, miRNAs have become a significant diagnostic and prognostic tool in recent years. The significance of miRNAs in the pathogenesis and progression of BC has revealed the potential relevance of aberrantly expressed miRNAs as biomarkers for the detection, diagnosis, classification and therapy of BC (53). First, the expression profile of miRNAs can be used to distinguish BC tissue from normal tissue. For example, the significant overexpression of miR-21, miR-106a and miR-155 and the decreased expression of miR-126, miR-199a and miR-335 have been reported in BC tissue when compared with normal tissue (54). A study has reported higher levels of miR-21, miR-10b and miR-31 in bilateral patients than in unilateral patients. Additionally, a number of miRNAs are differentially expressed in the luminal A, luminal B, basal-like, HER2^+^ and normal-like BC subtypes. For example, the upregulation of miR-17-92 clusters has been shown to account for the great distinction of triple-negative BC from other subtypes (55).

According to a study, there was a direct correlation between the expression levels of miR-10B, miR-21, and miR-335 in 112 breast tumor samples and disease-free survival as compared to healthy tissues (56). Additionally, miR-21 overexpression can be utilized as a prognostic indicator for BC lung metastases. It was also revealed in this study that overall survival and disease-free interval are correlated with downregulating the tumor suppressor miR-205. According to recent computational research, there is a direct correlation between worse survival rates for individuals with BC and the connection of miR-330-3p with MAF mRNA. Finding a meaningful predictive biomarker for BC may benefit from additional validation of these miRNAs (53, 57).

A study has identified miR-571, miR-139-3p, miR-206, miR-193a, miR-526b, miR-519 to be more than 1.5-fold downregulated in BC patients of age 50-53 (53). Patients with BC had higher blood plasma levels of miR-376-766c, miR-801, miR-148b, miR-424, miR-184, miR-409, miR-376a, miR-190, and miR-127-3p (58). Establishing whether the dysregulation of miRNA discovered in patient serum samples is tumor-derived is crucial. In both plasma and tumor tissues, eight miRNAs (miR-16, miR-21, miR-27a, miR-150, miR-191, miR-200c, miR-210, and miR-451) were up-regulated and miR-145 was down-regulated in patients with BC (58, 59). Further it was demonstrated that miR-451, miR-21 and miR-16 levels were significantly elevated in the serum of BC patients compared to the healthy individuals (60). Interestingly miRNA levels measured in the postoperative samples were drastically lowered when compared with preoperative samples (61). On study demonstrated that miR-155 levels are significantly higher in 81.9% BC patient samples and is an excellent diagnostic marker (**Table 1**). After surgical removal of breast tumor and four rounds of chemotherapy, 79% of patients exhibited reduced levels of miR-155 and sustained treatment response (75). Another study found that miR-18a, miR-181a and miR-222 showed the highest percentage difference in the serum samples of 205 women who eventually developed cancer and 205 women who remained cancer free (76). In the blood plasma elevated levels of miR-200a/b/c, miR-203 and miR-210 were also shown to have prognostic significance in BC patients (22).

**Table 1:** Circulating miRNAs as Diagnostic, Prognostic, or predictive biomarkers in BC.

MiRNA	Expression level	Diagnostic	Prognostic	Predictive	References
miR-125b	Higher expression in non-responsive patients	Yes	No	Yes	(62)
MiR-214	Discriminates malignant from benign tumors and healthy subjects	Yes	No	No	(63)
MiR-127-3p, -376a, -148b, -409-3p, -652 and -801	Higher in BC patients	Yes	No	No	(64)
MiR-484	Higher in BC patients	Yes	No	No	(63)
MiR-1246, -1307-3p, and -6861-5p	Higher in BC patients	Yes	No	No	(65)
MiR-4634 and -6875-5p	Lower in BC patients				
MiR-148b, -133a, and -409-3p	Higher in BC patients	Yes	No	No	(66)
MiR-21, -126, -155, -199a, and -335	Associated with histological tumor grade and sex hormone receptor expression	Yes	No	No	(67)
MiR-202 and let-7b	Higher expression in BC patients and correlates with tumor aggressive and overall survival	Yes	Yes	No	(68)
MiR-148b-3p and -652-3p	Lower in the BC patients	Yes	Yes	No	(69)
MiR-10b-5p	Higher levels correlate with poor prognosis				
MiR-18b, -103, -107, and -652	Associated with tumor relapse and overall	Yes	Yes	No	(70)
MiR-10b and -373	Higher in BC patients with LN metastasis	Yes	Yes	No	(71)
MiR-10b, 34a, and -155	Correlates with tumor stage and/or metastasis	Yes	Yes	No	(72)
MiR-21-5p, -375, -205-5p, and -194-	Higher in recurrent BC patients	Yes	Yes	No	(73)
MiR-34a, -93, -373, -17, and -155	Expression correlated with metastasis and HER2, PR, and ER status	Yes	No	No	(74)
MiR-155	Higher in BC patients; decreased level after chemotherapy	Yes	No	Yes	(75)

six major BC molecular subtypes (Luminal A, Luminal B, HER2 positive, basal-like (triple negative), normal-like and claudin-low). Circulating miRNAs were examined in relation to BC molecular subtypes in numerous studies. Numerous microRNAs that are discussed here were previously known to be dysregulated in cancerous cells and to contribute to the growth and metastasis of tumors. All things considered, differentiable criteria for comparisons were frequently taken into account when comparing cancer patients with different molecular features (e.g., HER2+ vs. HER2-; HER2+ vs. TNBC; Luminal vs. TNBC; Luminal vs. healthy controls; pre-therapy vs. post-therapy). The most current research is listed below and condensed in (**Table 2**).

**Table 2:** Circulating microRNAs identified in the different breast cancer subtypes.

MicroRNAs	Expression Level*/* Prognostic Value	Reference
miR-940	Down-regulated and predictor of worst TNBC prognosis	(77)
miR-34	Down-regulated in TNBC cases vs. healthy controls	(78)
miR-29a, -181a, -223, -652	Decreased level in Luminal A cases compared to healthy controls	(79)
miR-195, -145	Lower in TNBC compared to triple positive cases	(80)
miR-21-5p, -375, -205-5p, -194-5p	Up-regulated in HR+ and TNBC recurrent patients	(52)
376c-3p, -411-5p	Down-regulated in HR+ and TNBC recurrent patients	(81)
miR-195	Decrased level in post-surgery Luminal A and B cases.	(82)
	Significant increase in pre-operative Luminal B	
miR-21	Higher in non-metastatic HER2+ compared to HER2- cases	(82)
miR-373	Higher in TNBC compared to Luminal BC and in HR- compared to HR+	(83)
miR-15a	Lower level in HER2+ vs. HER2- cases of inflammatory BC	(84)
miR-18b, -103, -107, -652	Predictors of tumor relapse and OS in TNBC	(85)

For early-stage breast cancer, surgery is currently the main course of treatment. Nonetheless, survival rates have significantly increased due to the use of hormone treatment, radiation, and chemotherapy (86). Chemotherapy and radiotherapy are utilized as neoadjuvant treatments for metastatic breast cancer, prior to mastectomy or breast conservative surgery and axillary node clearance (87). Chemotherapy, radiation therapy, and hormone therapy are the main treatments for progressing disease (stage IV). Identifying metastatic particular miRNA may help classify breast cancer and guide and enhance treatment decisions (6). Additionally, the application of certain miRNA as therapies may prove to be a useful therapeutic approach in the future. In fact, there are ongoing clinical studies examining the effectiveness of miRNA therapy for cancer (88).

Chemotherapy is the most common treatment for metastatic breast cancer and typically entails a mix of medications, frequently in conjunction with hormone therapy. The most often utilized chemotherapeutics include cyclophosphamide, taxanes (paclitaxel and docetaxel), anthracyclines (doxorubicin and epirubicin), and fluorouracil (5-FU) (89). Notably, the median survival rate for HER2+ve patients receiving trastuzumab in addition to chemotherapy rose from 20.3 to 25.1 months. Even with these advances in treatment, a significant number of patients remain unresponsive to conventional chemotherapy or hormone therapy. Circulating miRNAs have been investigated in this context as putative biomarkers to forecast treatment response (90). Just a small number of recent research have looked into how miRNAs relate to treatment that is subtype-specific. Better patient outcomes will result from the early detection of circulating miRNA that can diagnose illness and/or chemotherapeutic responses. This would considerably simplify improved treatments (91).

Biomarkers that predict treatment success play a critical role in the management of BC therapy (92). According to a particular idea, which is one may anticipate how well patients with BC will respond to treatment by looking for certain miRNAs that are dysregulated in BCs (93). Numerous miRNAs have been found to have a significant role in BC thus far, making them desirable targets for therapeutic intervention. Several research groups have demonstrated how to regulate this target miRNA’s expression to improve therapy. MiRNA-based therapy can be used to treat cancer in two different ways: either by increasing the production of tumor suppressor miRNAs or by blocking oncogenic miRNA (94). Two important miRNA therapeutics are oligonucleotide analogs and antagonists. By employing single-stranded oligonucleotides containing miRNA-complementary sequences, target protein miRNA function is inhibited. Functional miRNA liposomes have been developed in TNBC cells to inhibit the TGF-β1/SMAD pathway and SLUG expression, thereby enhancing the efficacy of chemotherapy in mice. In order to treat BC, epigenetic methods such as functionally cooperative miRNAs or histone deacetylase inhibitors are useful for blocking HER3 signal transduction (95). Moreover, 2,3,7,8-tetrachlorodibenzo-p-dioxin (TCDD) therapy results in the elevation of circulating miRNA through miR-3942-3p/BARD1, which inhibits the cell cycle and promotes apoptosis to end BC (96).

miRNA analogs are used to restore miRNAs that have lost function, much like traditional gene therapy, while miRNA antagonists can block the function of miRNAs in human disease. Another name for this strategy is miRNA replacement treatment (94). More people are interested in this novel treatment since it might present a fresh chance to create tumor inhibitors. Furthermore, it has been possible to increase the transmission efficiency of miRNA or anti-miRNA molecules *in vivo* by using anti-miRNA oligonucleotides (AMOs), locked nucleic acid (LNA)-modified oligonucleotides, cholesterol-binding anti-miRNA molecules, and 2′-O-methoxyethyl-4′-sulfur RNA (MOE-SRNA) (97 *)*. Long half-lives and high efficiency modified miRNA molecules have also been created. To increase medication effectiveness, miRNA mimics or antagonists were incorporated into the packaging of nanomaterials, particularly gold nanoparticles. A novel nanoplatform called DTX (docetaxel)/miR-34a nano carrier combines insoluble medications with gene/protein medications, offering a potentially effective treatment for metastatic BC (97).

Several tactics are being employed to inhibit the metastatic and carcinogenic potential of miRNA. One of the most obvious strategies is to quiet these oncogenic miRNAs and prevent them from interacting with their target proteins by using an anti-miRNA oligonucleotide (AMO). By competing with their target mRNA, the AMO stops oncogenic miRNAs from accomplishing their intended purpose (98). Anti-miRNA 2-*O*-methyl or locked nucleic acid oligonucleotides used to inactivate oncomirs such as miR-21 in breast tumors may taper tumor growth. Anti-miR-21-induced reduction in tumor growth to be potentiated by the addition of the chemotherapeutic agent topotecan, an inhibitor of DNA topoisomerase I. This suggests that suppression of the oncogenic miR-21 could sensitize tumor cells to anticancer therapy, which is an exciting prospect for patients exhibiting a poor response to primary chemotherapy (7).

Another novel approach in miRNA antagonism is by using peptide nucleic acid (PNA) as a miRNA inhibitor. Chemically in PNA the sugar-phosphate backbone is replaced by N-(2-aminoethyl) glycine, which makes them efficient hybridization agent resistant to DNAses and proteases. In aggressive BC cells PNAs targeting miR-221 and miR-210 reduced the levels of miR-221 and miR-210 respectively. Also, inhibition of these miRNAs resulted in the elevated levels of a major apoptosis player p27Kip1 (53). Treatment with PNA targeting miR-21 showed inhibition of tumorigenesis of MCF-7 BC cells in female nude mice compared to the control group of mice (99).

The induction of tumor suppressor miRNA expression using viral or liposomal delivery of tissue-specific tumor suppressors to affected tissue may result in the prevention of progression, or even shrinking, of breast tumors. Tumor suppressor miRNA induction has also been shown to be subject to epigenetic control. Using chromatin remodeling drugs to simultaneously inhibit DNA methylation and histone deacetylation, epigenetic alterations in cancer and normal cells. The miRNAs were upregulated in tumor cells but not in normal cells. MiR-127, which exhibited reduced expression in 75% of human cancer cells tested, was significantly upregulated after treatment. The induction of this miRNA was associated with downregulation of the proto-oncogene BCL6, suggesting a cancer-protective effect for miR-127 and a novel therapeutic strategy for the prevention and treatment of malignancy. This concept of inducing tumor suppressor miRNA expression has been termed “miRNA Replacement Therapy”; in anticipation of the promising clinical potential it. Several tumor suppressor miRNAs are shown to be downregulated in various types of cancers. It is proposed that restoring their levels should be beneficial in inhibiting cancer progression. One approach to restore the tumor suppressor miRNA in cancer cells is by using the miRNA mimics. A study demonstrated that in aggressive BC cell MDA-MB-231 restoring the levels of miR-200c using the miR-200c mimic results in reduced cell proliferation, invasion and migration (100). Similarly, in BC cells increasing the levels of let-7 miRNA by let-7-lentivirus infection reduced the cell proliferation and mammospheres formation (101).

## 7 Circulating miRNA as biomarkers in metastatic BC

BC metastasis is a process described as spread via regional lymph nodes to distal and proximal tissues such as the bones, lungs, brain, and liver. Indeed, the rates and sites of distal metastasis can vary depending on age and stage of diagnosis (102). The process of metastasis is a series of events that arises from the evolutionary acquisition of traits that enable tumor cells from one primary site to migrate, disseminate, dissociate, survive, extravagate, and infiltrate a distant site to form a metastatic niche. This allows the tumor cells to continue to exist and multiply in a new environment (103). Circulating tumor cells (CTCs) are important in the process of spreading tumor cells from the original location into the circulation. In order to infiltrate the extracellular matrix at the primary location, these cells go through EMT. Primary tumor cells and CTCs eventually release RNAs and miRNAs condensed in exosomes to withstand anoikis and immunosurveillance, which aids in the survival of metastatic cancer cells at the metastatic site (104). Furthermore, cellular adaptability is essential for invasion and colonization of secondary tumor sites. This is supported by various signaling pathways, such as the EGFR, OPG/RANK/RANKL, TGF-β, IGF system, PI3K/Akt/mTOR, Wnt, Hippo, HIF-1, TNF, FoxO, JAK/STAT, PD-1/PD-L1, and epithelial-mesenchymal transition (EMT) pathways, among others (105). It is crucial to emphasize that a number of miRNA molecules control various pathways, emphasizing how crucial they are to the illness. miRNA circulating to be used as metastatic BC indicators. Using both mouse and human cells, miR-10b was the first miRNA to be demonstrated to be substantially expressed in metastatic BC and to have a clinical correlate with primary BCs (106). It was shown unequivocally that treatment with AMO against miR-10b markedly reduced the levels of miR-10b while concurrently increasing the levels of HOXD10, the target of miR-10b. Furthermore, it was noted that anti-sense miR-10b prevented lung metastasis but did not shrink the size of the original tumor (53). These results suggest that anti-miR-10b can be a good candidate as an anti-metastatic agent but might not be useful in reducing primary tumor burden in BC (53). In subsequent study, it was discovered that patients with metastatic BC had higher levels of miR-10b, miR-34a, and miR-155. Most recently, it was demonstrated that lymph node-positive BC was associated with elevated levels of miR-10b and miR-373 (107). It is noteworthy that patients with lymph nodes showed a markedly higher level of circulating miR-10b and miR-373 than either healthy controls or patients without nodal involvement. Subsequent research also revealed differences in the miRNA levels between lymph node positive and negative patients, with the former showing greater levels of miR-20a and miR-214. But only in a limited sample did miR-210 emerge as a putative indicator of lymph node metastases (108). Remarkably, miR-10b was found to be a putative biomarker for BC metastases to the brain and bones. However, taken as a whole, these disparate findings raise questions about the efficacy of miR-10b as a marker specific to metastases. Additionally, it was discovered that individuals with metastatic BC differed significantly in miR-299-5p and miR-411, with the additional miRs miR-215 and miR-452 showing potential but not achieving statistical significance (109). Furthermore, miR-21 has been found to be a marker and stage predictor for BC. In patients with circulating tumor cells (CTC), eight miRNAs (miR-141, miR-200a, miR-200b, miR-200c, miR-203, miR-210, miR-375, and miR-801) were shown to be significantly increased recently (110). Targeting several tumor/metastasis suppressor genes, including PDCD4, PTEN, and TIMP3, miR-21 contributes to invasion and metastasis, indicating its potential as an anti-metastasis therapeutic. It has been demonstrated that overexpressing miR-145 inhibits the proliferation and motility of BC cells, and a treatment that combines Ad-miR-145 with 5-FU has strong anticancer effects. Additionally, miR-146 suppresses metastasis; in comparison to control cells, those expressing miR-146a/b exhibit significantly reduced invasion and migratory capabilities (111).

In BC cells and tissues, miR-31 has also been demonstrated to control several genes linked to metastasis, and the expression of this gene is negatively correlated with the cell’s capacity for invasion and metastasis. When miR-31 is activated in metastases that have already formed, metastatic regression occurs and patient survival is extended. In a different study, early-onset breast tumors that metastasized had higher levels of miR-105 than malignancies that did not. Additionally, this study discovered that miR-105 overexpression encouraged metastasis *in vivo*. It has been determined that the miRNAs miR-17 and miR-155 may distinguish between BC that has spread to other organs and non-metastatic BC (112). Numerous of these miRNAs have been found to be markers in metastatic triple negative BC samples, which lends support to those investigations. The fact that the metastasis sites were not mentioned in detail in the aforementioned papers could partially explain the variety of miRNA found. These results, which are significant, emphasize the value and possible uses of circulating miRNA as biomarkers that can distinguish between BCs that are not metastatic and those that are (113).

## 8 AI-based strategies for miRNA use in BC

Microarray and Next-Generation Sequencing (NGS) are examples of high-throughput technologies that allow for the simultaneous profiling of hundreds of miRNAs in BC samples. NGS has made it possible to examine the expression levels of numerous miRNAs in various cancers and investigate their association with cancer development and progression. However, it might be challenging to distinguish true correlations between miRNAs and cancer from accidental links due to the abundance of miRNAs and patient variances (114). Artificial Intelligence (AI) is a viable way to get beyond these challenges. Artificial intelligence (AI) is a sophisticated technology that recognizes complicated healthcare unit difficulties using mathematically based algorithmic concepts that are akin to those of the human mind. These methods may effectively extract significant characteristics and assist in identifying miRNA signatures, predicting targets, and predicting tumor-specific biomarkers by integrating and evaluating large datasets from various sources (115). AI can improve the miRNA biomarker finding process. AI systems have the ability to use clinical factors and miRNA expression data to forecast treatment response and pinpoint possible therapeutic targets. Oncologists can use this information to customize treatment regimens for increased effectiveness and fewer adverse effects (116).

However, miRNA–mRNA interaction networks can also be analyzed using AI algorithms, which can help decipher the intricate regulatory networks underlying BC pathogenesis. AI can thus aid in our understanding of the intricate regulatory networks involved in the pathophysiology of BC (117).

The application of AI to miRNA analysis in cancer is a promising field of study, despite its difficulties. With larger miRNA expression datasets becoming available and AI technology continuing to progress, AI is expected to become more crucial to the diagnosis, prognosis, and therapy of cancer (117). The near future should see improved integration of AI into normal BC management because to advancements in AI algorithms and more collaboration between researchers, doctors, and industry stakeholders (118).

## 9 Conclusion

In this review, we reviewed that miRNAs play important roles initiation and progression of human malignancy holds great potential for new developments in current diagnostic and therapeutic strategies in the management of patients with BC. Our review indicates that miRNAs may be used for therapeutic purposes, prognosis, and clinical diagnosis. As a result, miRNAs are emerging as novel biomarkers for BC diagnosis and prognosis, and they may also be able to predict how a tumor will react to a particular chemotherapy treatment. Thus, a compelling case for the clinical use of miRNA biomarkers may be made by these published investigations. Numerous factors, including as variations in sample preparation, small sample sizes, distinct miRNA experimental techniques (qPCR or distinct miRNA array platforms), and treatment/tumor heterogeneity, impact the data presented by the various research. It will take additional research on more homogeneous populations to pinpoint these miRNAs for the diagnosis of BC. AI for miRNA analysis in cancer is a promising area of research. With continued advances in AI technology and the availability of larger miRNA expression datasets, AI is likely to play an increasingly important role in cancer diagnosis, prognosis, and treatment.

## Author contributions

TB: Conceptualization, Data curation, Formal analysis, Funding acquisition, Investigation, Methodology, Project administration, Writing – original draft, Writing – review & editing. MK: Supervision, Visualization, Writing – review & editing. MG: Funding acquisition, Methodology, Resources, Supervision, Writing – original draft. GG: Data curation, Formal analysis, Investigation, Writing – original draft. MJ: Formal analysis, Funding acquisition, Methodology, Resources, Supervision, Writing – original draft. HA: Formal analysis, Funding acquisition, Investigation, Writing – review & editing. AN: Conceptualization, Formal analysis, Investigation, Writing – review & editing. MB: Resources, Supervision, Validation, Visualization, Writing – original draft.
